# Supplementary material for: Analyzing Strawberry Preferences: Best–Worst Scaling Methodology and Purchase Styles
Source: Foods. 2024 May 10;13(10):1474. doi: 10.3390/foods13101474 (PMC11120401; doi:10.3390/foods13101474)
Supplement: Supplementary file 1 [file foods-13-01474-s001.zip › foods-2953231-supplementary.pdf]

Survey on the perception of consumers towards strawberries

Dear interviewee,

We are a team of researchers specializing in the field of food, with a particular focus on strawberries. Our initiative involves conducting a survey to analyze consumer behavior and food habits related to strawberries. Our objective is to investigate preferences and consumption patterns, considering individual attitudes toward strawberries.

The questionnaire that is structured as follows:

1) in the first section some of your socio-demographic characteristics will be collected;

2) in the second section the importance towards strawberries and consumption habits;

3) in the third and last section questions was dedicated to the assessment of the consumers' declared preferences pertaining to 12 strawberry attributes.

The compilation will take you 5-6 minutes maximum and you are free to answer the full questionnaire or refuse to fill it out at any time you wish.

The data collected are totally anonymous and will not be used for profit.
Feel free to abandon the questionnaire.

Thank you for your cooperation

> I agree to participate in the research study. I understand the purpose and nature of this study and I am participating voluntarily. I understand that I can withdraw from the study at any time, without any penalty or consequences.
> I understand that all the information acquired by you will be anonymous and will follow the EU DGPR n. 2016/679 and that any eventual comments or opinion provided will be maintained confidential and I grant permission for the data generated from this interview to be used and processed for the purposes of the research in compliance with all applicable laws and regulations, including in publications arising from it.
> I understand that data will be processed in compliance with all applicable laws, regulations and ethical standards.

Date	Signature
______________	_________________________

For any questions: Antonina Sparacino, Department of Agricultural, Forestry and Food Sciences, University of Turin, Largo Braccini 2, 10095 Grugliasco, Italy (antonina.sparacino@unito.it)

Survey on the perception of consumers towards strawberries

Section 1. Socio-demographic features

1) Gender
   - Female
   - Male
   - No answer
2) Age
   - 18-25
   - 26-35
   - 36-45
   - 46-55
   - 56-65
   - Over 65
3) Zip code of residence ___________
4) Household composition
   - 1 component
   - 2 components
   - 3 components
   - 4 components
   - 5 components or more
5) Number of school-aged children
6) Educational level
   - Primary school
   - Lower secondary school
   - Upper secondary school
   - Degree/post-graduate
7) Employment situation
   - Student
   - Employed
   - Self-employed
   - Retired
   - Seeking employment
   - Housemaker
8) Annual income range
   - <25,000
   - 25,000-40,000
   - 40,000-60,000
   - >60,000
   - No answer

Section 2. Strawberry consumption habit

1) Where do you usually buy fresh strawberries?
   - Greengrocer
   - Open-air market

For any questions: Antonina Sparacino, Department of Agricultural, Forestry and Food Sciences, University of Turin, Largo Braccini 2, 10095 Grugliasco, Italy (antonina.sparacino@unito.it)

- Organic shop
- Directly from the producer
- Super/hypermarket or discount
- Grocery store
- Ethical purchasing groups (GAS)

2) In which season do you buy and consume strawberries?
   - Spring
   - Summer
   - Autumn
   - Winter
   - Over the entire year
3) How often do you consume strawberries?
   - 2-3 times a week
   - One/twice a week
   - Less than once a week
   - Once a month
   - Less than once a month

Section 3. Consumers' preferences assessment

Imagine you are purchasing strawberries.

For each set of 4 attributes, select a single option influencing THE MOST your choice and a single option influencing THE LEAST your choice.

Attribute list differently combinate for the 4 version of the survey:

- Brand
- Indication of origin (national/ foreign)
- Price
- Packaging
- Offers
- Appearance
- Taste/Aroma
- Health benefits
- Local origin
- Organic certification
- Quality certification
- Seasonality

For any questions: Antonina Sparacino, Department of Agricultural, Forestry and Food Sciences, University of Turin, Largo Braccini 2, 10095 Grugliasco, Italy (antonina.sparacino@unito.it)
